# Rapid and accurate taxonomic classification of cpn60 amplicon sequence variants

**DOI:** 10.1038/s43705-023-00283-z

**Published:** 2023-07-21

**Authors:** Qingyi Ren, Janet E. Hill

**Affiliations:** Department of Veterinary Microbiology, University of Saskatchewan, Saskatoon, SK Canada

**Keywords:** Microbiome, Sequencing

## Abstract

The “universal target” region of the gene encoding the 60 kDa chaperonin protein (cpn60, also known as groEL or hsp60) is a proven sequence barcode for bacteria and a useful target for marker gene amplicon-based studies of complex microbial communities. To date, identification of cpn60 sequence variants from microbiome studies has been accomplished by alignment of queries to a reference database. Naïve Bayesian classifiers offer an alternative identification method that provides variable rank classification and shorter analysis times. We curated a set of cpn60 barcode sequences to train the RDP classifier and tested its performance on data from previous human microbiome studies. Results showed that sequences accounting for 79%, 86% and 92% of the observations (read counts) in saliva, vagina and infant stool microbiome data sets were classified to the species rank. We also trained the QIIME 2 q2-feature-classifier on cpn60 sequence data and demonstrated that it gives results consistent with the standalone RDP classifier. Successful implementation of a naïve Bayesian classifier for cpn60 sequences will facilitate future microbiome studies and open opportunities to integrate cpn60 amplicon sequence identification into existing analysis pipelines.

## Introduction

High throughput, massively parallel sequencing of PCR amplified marker gene sequences (barcodes) remains an important technique for determining the taxonomic composition of complex microbial communities. While variable regions of the 16 S rRNA gene remain the most widely targeted markers for bacteria, high resolution microbiome profiling has also been achieved using partial cpn60 sequences; an approach that offers some particular advantages. The cpn60 “universal target” region (corresponding to nucleotides 274–828 of the *E. coli* cpn60 gene, also known as hsp60 or groEL) has been demonstrated using the International Barcode of Life framework to be a preferred barcode for bacteria [1], with greater inter- and intra-species distances than variable regions of the 16 S rRNA gene. Due to its conservation in bacteria and eukaryotes (and some archaea), cpn60 barcode sequencing can provide simultaneous detection of bacteria and fungi in microbial communities [2]. cpn60 barcode sequences are excellent predictors of whole genome sequence relationships [3–5] and have been used for sub-species resolution of bacterial taxa [6–9]. The availability of “universal” PCR primers [10, 11] targeting the cpn60 barcode and a manually curated database of reference sequences (cpnDB [12]) have made this sequence an attractive target for diagnostics [13–15], characterization of new bacterial species [16–18], the assessment of bacterial contamination in virome studies [19] and taxonomic profiling of a variety of microbial communities, including microbiota of human and animal body sites [20–25], and environmental samples [26–28].

Regardless of the marker gene targeted in an amplicon sequencing study, taxonomic identification of the resulting sequence requires a reference database appropriate for the environment under investigation and a robust method for sequence comparison that results in accurate identification. A wide range of alignment methods have been developed for comparing microbiome derived sequences to reference databases. For alignment of cpn60 barcode sequences amplified from microbiomes, wateredBLAST [29] was developed. wateredBLAST uses a combination of BLASTn and Smith-Waterman alignments and results in the identification of the “nearest neighbor” in the reference database for each query based on percent sequence identity over the entire length of the query. A limitation of this approach, and any other alignment-based method, is that only species level identification is reported even if the percent identity is low; two amplicon sequences with different levels of identity to the same nearest neighbor end up with the same identification. This potential for false positive results is an even larger issue for alignment of data from environments whose constituents are not well represented in the reference database. Another disadvantage of alignment methods in general is that they can be slow and expensive in terms of computational resources, requiring large amounts of memory. This is increasingly problematic as microbiome studies expand in size and scope, with a corresponding increase in the amount of sequence data produced.

Sequence classifiers offer an alternative to alignment methods and have two potential advantages: they tend to be faster since they reduce queries and databases to unique “words” (kmers), and they allow variable rank classifications since taxonomic lineage information is included in the reference data. Users can decide which ranks to accept based on the confidence value associated with each result. Several different classifiers have been developed with the naive Bayesian classifier, RDP Classifier [30], being perhaps the most widely used. The RDP Classifier was originally designed for 16 S rRNA data from prokaryotes, but if training data is available, it can be used for other targets, such as large-subunit RNA (LSU) in fungi [31] cytochrome oxidase subunit 1 (CO1) in animals [32], and rbcL and ITS2 sequences in plants [33]. The success of sequence classification depends upon the availability of high-quality reference data with complete taxonomic lineage information. Incorrectly annotated reference sequences can lead to false positive classification results, while gaps in coverage of the reference database relative to the environment under study can lead to failure to classify beyond the highest taxonomic ranks.

The cpnDB reference database is manually curated and focuses on incorporation of bacterial type strain data to ensure robust landmarks for sequence identification [12]. The establishment of amplicon sequence variant (ASV) calling for cpn60 barcode sequences and the demonstration that as little as 150 bp from the 5’ end of the barcode region can provide species level identification [34] have resulted in improvements to analysis of cpn60 sequence data from microbiome studies, however, reliance on alignment methods for taxonomic identification remains a limitation that has presented a barrier to uptake of cpn60 barcode sequencing approaches. The objectives of the current study were to investigate the accuracy of the RDP classifier for taxonomic assignment of cpn60 sequences from a curated reference database, to compare RDP classifier and wateredBLAST taxonomic assignments of ASV data from human salivary, vaginal, and infant stool microbiomes, and to provide trained cpn60 sequence classifiers to the research community.

## Materials and methods

### Generation of training files

The RDP classifier (version 2.13) provided within RDPTools was used in this study. All group I reference nucleotide universal target (UT) sequences in the cpnDB reference database (accessed May 2022) were used to generate the training files. From the reference database, the name of each taxon (genus+species) and cpn60 UT sequence (fasta files) were obtained. The full taxonomic lineage for each taxon was obtained from the NCBI taxonomy database using the *taxarank* function in taxonomy_ranks [35, 36]. For each taxon, any taxonomic rank without a corresponding entry returned a result of “NA”, which was replaced with “-” to make the resulting taxonomy table interpretable in subsequent steps. For any taxon that returned more than one *taxarank* result (e.g. where a eukaryote and a prokaryote share the same genus+species name), the eukaryotic lineage was then manually deleted from the taxonomy table. Similarly, taxa without a lineage that extended to the species level were removed from the taxonomy table and from the sequence data file. Python scripts (*Lineage2taxTrain* and *addFullLineage*) for creating “ready to train” taxonomy table and fasta files were obtained from https://github.com/GLBRC-TeamMicrobiome/python_scripts. The *Lineage2taxTrain* python script was then applied to the modified taxonomy table to produce the “ready to train” taxonomy file. For the fasta files, the *rm-dupseq* function of the classifier was used to remove any duplicate sequences (since these can inflate results during classification performance testing), then the *addFullLineage* script was used to insert lineage information into the definition line of each sequence. Finally, *Classifier.jar* was used to generate the training files from the two ready to train files, and the rRNAClassifier.properties file was downloaded from https://github.com/rdpstaff/classifier/blob/master/samplefiles/rRNAClassifier.properties and added in the output directory.

### Testing and evaluating the performance of the classifier

Initial testing of the trained classifier was performed by using the training sequences as queries and evaluating the results in terms of whether sequences were correctly classified as themselves.

Cross-validation (also known as leave-one-out testing) was performed to evaluate the accuracy of the classifier using the built-in leave-one-out command within the RDP classifier with the default confidence cutoff of 0.9. Classifiers trained on different 5’-anchored lengths of cpn60 barcode sequence (50 bp, 150 bp, 300 bp, 450 bp and full length) were used in cross-validation, and the accuracy of results for each query length were compared.

### Classification of microbiome sequence data and comparison with wateredBLAST

ASV sequences (150 bp) from three previously collected microbiome data sets (saliva (6737 ASV from 89 samples) [37], vagina (1950 ASV from 283 samples) [38], and infant stool (4153 ASV from 584 samples) [39]) were analyzed using the trained classifier with the default confidence value of ≥0.8 to assign a rank. To evaluate the accuracy of the classifier results, the same queries were identified using wateredBLAST for comparison. The same set of cpn60 sequences used to train the classifier was used as the reference database for wateredBLAST. Results from wateredBLAST and the classifier were compared based on species level identification/classification results and time taken for the analysis. Species rank confidence and wateredBLAST percent identity results for each sample type were visualized XY density plots using geom_density_2d and ggplot2 in R (version 4.2.1).

To test whether having the query the same length as the sequences in the training set influenced the classification results, the classifier was trained on the reference data set trimmed to the first 150 bp. The stool microbiome ASV sequences (150 bp) were then analyzed using either the Classifier trained on 150 bp or full-length (~555 bp) cpn60 barcode sequences and results were compared using Spearman’s correlation test in GraphPad Prism v 9.5.0.

All classifier results were parsed with a custom python script to extract confidence values for each taxonomic rank for analysis.

### Establishing classifier thresholds for identification of non-cpn60 sequences

To determine a recommended cutoff (taxonomic rank and confidence level) for the classifier to filter out non-cpn60 sequences, two values were chosen for further assessment based on visual inspection of initial results: 0.6 and 0.8 at the phylum level. In order to choose which one was a better indicator of non-cpn60 sequences, specificity and sensitivity were calculated using wateredBLAST as the gold standard since it has been demonstrated that the minimum identity of cpn60 sequences to any cpnDB reference sequence is 55% [40]. Sensitivity was calculated as: true positive/(true positive + false negative) and specificity was calculated as: true negative/(true negative + false positive).

### Implementation of the QIIME2 q2-feature-classifier

To demonstrate that cpn60 ASV classification can be integrated into a QIIME 2 workflow with the q2-feature-classifier plugin [41], we generated an appropriately formatted taxonomy table, created a classifier artifact (trained a classifier with the fit-classifier-naive-bayes option) in QIIME 2 [42] (v2022.2), classified the vaginal microbiome ASV, and compared the results to the standalone RDP classifier results.

## Results and discussion

### Training the classifier

The cpnDB reference database of 17,815 sequences was selected as the initial training data. After removing duplicates and errors, 17,778 entries were included. The *taxarank* script was then applied to obtain the full taxonomic lineage of all taxa, which generated 17,793 entries in the resulting taxonomy table because some taxa yielded multiple entries. Duplicate entries were removed manually, leaving 17,778 entries. Taxa that were not labeled to the species level (e.g., *Staphylococcus* sp.) were removed leaving 16,416 entries. The *rm-dupseq* function of the RDP Classifier was used to remove duplicate sequences in the training set, resulting in 11,001 unique sequences to train the classifier. This is obviously much smaller than commonly used 16 S rRNA training sets, such as the Silva database, which contains 128,884 bacterial, 2846 archaeal, and 14,871 eukaryotic sequences as of release 138.1 [43]. Even though cpn60 training set has fewer sequences, it has substantial taxonomic breadth (2096 genera) as a result of the cpnDB curation strategy, which emphasizes broad coverage of taxa rather than many redundant entries for individual species [12].

### Initial testing of the classifier

After training, the functionality of the classifier and the validity of the training files were tested by using the training set as queries. The training set contained 11,001 sequences, and 9849 (89.5%) of these were classified to the species rank with a confidence level of 1.0 (i.e., they were classified as themselves as expected). Out of the remaining 1152 sequences, 799 had a confidence value of 0.8–0.99 at the species rank. Out of the 353 sequences which were not classified confidently at the species rank, 321 (90.9%) were classified at the genus rank, a result expected to occur in cases where there are multiple very similar sequences within a genus thus lowering confidence beyond that level.

### Cross-validation

**Fig. 1:**
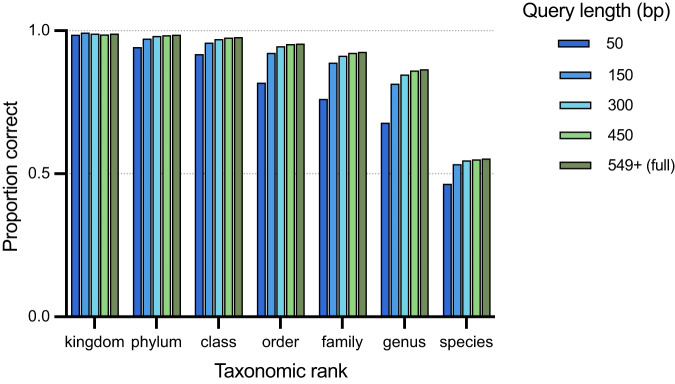
Effects of sequences lengths on cross-validation results. Five different 5' anchored lengths of the same set of 11,001 sequences were assessed.

In cross-validation, each sequence is removed in turn from the training set and then classified based on the remaining sequences. To determine the effect of sequence length on classification accuracy, cross-validation was performed with 5' anchored sequence lengths of 50, 150, 300, 450 bp and full-length (~555 bp) cpn60 barcode (Fig. 1). Regardless of length, >90% of queries were accurately classified to the ranks of kingdom, phylum and class. At lower taxonomic ranks, the 50 bp sequences were classified markedly more poorly than longer sequences.

An approximately 30% of drop in the proportion of accurately classified sequences was observed at the species rank relative to genus rank. Any species that was unique in the training set could obviously not be correctly classified. Accurate species level classification was only possible in cases where there were multiple strains of individual species with non-identical cpn60 sequences in the training set. In some cases, the complete database did contain multiple records for a species but if the cpn60 sequences were identical they were removed by the remove-duplicate step in preparing the training files. If there were multiple entries for a species with non-identical cpn60 sequences in the training set, accurate species level classification was possible, which was the case for just over half of the queries in the cross-validation experiment (Fig. 1).

Sequence lengths of ≥150 bp all provided accurate results at the genus rank for at least 80% of queries, and the proportion of accurately classified sequences increased as the query length increased (Fig. 1). As expected, the proportion of accurately classified sequences also increased at higher taxonomic ranks. Improved accuracy with longer query lengths was expected given the nature of a naive-Bayesian classifier, where longer sequences generate more “words” for the classifier to look up. However, the improvement in accuracy for lengths beyond 150 bp was minimal. This observation is consistent with previous work showing that using alignment methods, 150 bp from the 5' end of the barcode region is sufficient for unambiguous identification [44].

### Classification of human microbiome sequence data

**Fig. 2:**
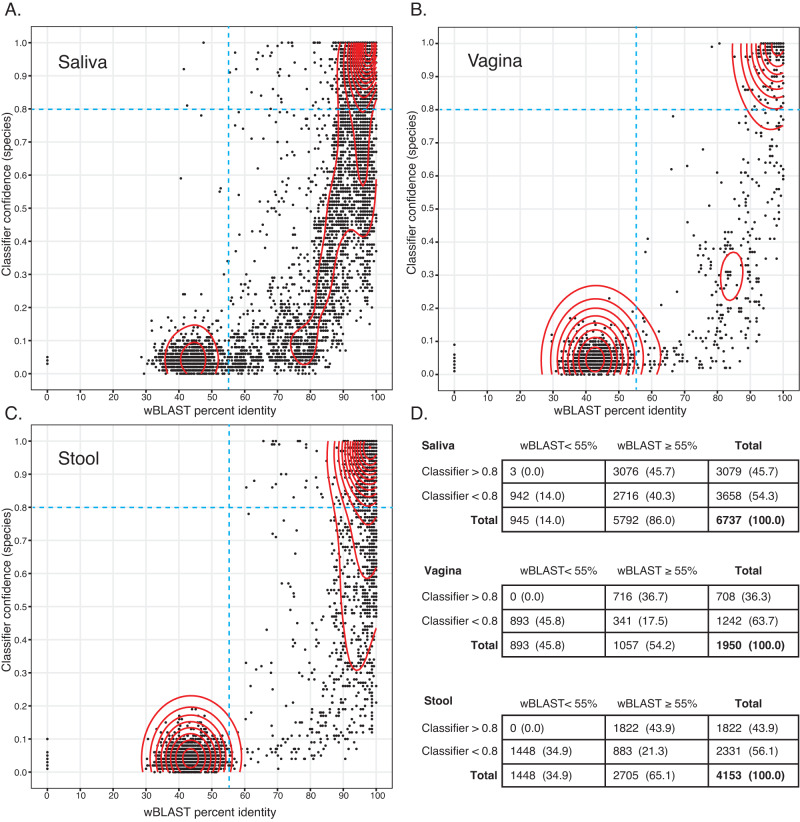
Comparison of wateredBLAST and classifier results for cpn60 ASV sequences from human microbiomes. Taxonomic identification of 150 bp cpn60 ASV from saliva (**A**), vagina (**B**) and infant stool (**C**) microbiomes by wateredBLAST (abscissa, % identity) and the classifier (ordinate, confidence at species rank). Density of data points is indicated by red contour lines. Broken blue lines indicate two thresholds: ≥55% sequence identity for wateredBLAST (the established cut-off for non-cpn60 sequences) and 0.8 for the classifier (the default confidence threshold). Number (percent) of ASV in each quadrant of the plots are shown in (**D**).

To compare the classifier and wateredBLAST for species level identification of cpn60 ASV from microbiomes, we analyzed ASV sequences from previously conducted studies focused on salivary, vaginal and infant stool microbiomes. ASV sequences were aligned to the training sequences with wateredBLAST or identified using the classifier with results plotted as percent identity to a reference sequence or confidence at species rank, respectively (Fig. 2). ASV identification was more rapid with the classifier than wateredBLAST for each set of queries: stool (28.1 s vs. 86.4 s), vagina (49.3 s vs. 198.1 s) and infant stool (72.8 s vs. 323.6 s).

We divided the x-y plots into four quadrants based on two values: ≥55% for wateredBLAST and ≥0.8 for the classifier (Fig. 2). The left two quadrants consist of sequences that have a sequence identity of <55%, which according to our past experience, are not cpn60 [40]. As expected, queries with wateredBLAST scores of <55% had correspondingly low classifier confidence values at the species rank, generally <0.2 (Fig. 2). Interestingly there were three queries in the salivary microbiome data that gave wateredBLAST scores <55% but were classified confidently (>0.8) at the species level (Fig. 2A). Despite the low wateredBLAST identities, the two methods agreed on the identification of these three ASV as *Rothia mucilaginosa, Selenomoas infelix* and *Centipeda periodontii*.

The right top quadrant in each plot in Fig. 2 includes ASV that are cpn60 based on the ≥55% wateredBLAST cutoff criterion and are confidently classified at species rank by the classifier. For all microbiome data sets this quadrant accounts for the majority of ASV that met the wateredBLAST criterion to be identified as a cpn60 sequence (3076/5792 (53.1%), 716/1057 (67.7%), 1822/2705 (67.4%) for saliva, vagina and stool, respectively) (Fig. 2D). Additionally, for the majority of these ASV, identifications by wateredBLAST and the classifier were in agreement. Within the upper right quadrant, most ASV were actually ≥90% identical to their nearest reference sequence by wateredBLAST (2931/3079 (95.2%), 705/708 (99.6%), and 1780/1822 (97.7%) of saliva, vagina and stool, respectively). Within these ASV, 2735/2931 (93.3%), 683/705 (96.9%), and 1617/1780 (90.8%) of saliva, vagina and stool ASV, respectively, had equivalent species identification results from wateredBLAST and the classifier. In cases where the wateredBLAST and classifier species results did not agree there was agreement on genus, although in a handful of cases there disagreement was related to a recent name change (e.g., *Atlantibacter* vs. *Escherichia*).

The lower right quadrants of the density plots in Fig. 2 contain ASV queries that are cpn60 (≥55% identical by wateredBLAST) but are not confidentially classified at the species rank by the classifier. These sequences were, however, classified confidently at higher ranks. 1024/2716 (37.7%) of these lower right quadrant ASV sequences from the saliva microbiome, 56/341 (16.4%) ASV sequences from the vaginal microbiome, and 435/883 (49.3%) ASV sequences from the infant stool microbiome were classified confidently at the genus rank and the remainder were classified at higher ranks.

To identify factors that contribute to a high wateredBLAST percent identity and a relatively low species rank classifier confidence, we examined results for saliva microbiome query sequences that were: identified with a percent identity of ≥90% by wateredBLAST and classified with a confidence level of <0.8 at species rank by the classifier. These queries were further divided into two categories: those that were classified with a confidence level of ≥0.8 at genus rank by the classifier (query type A, 887 ASV), and those that were classified with a confidence level of <0.8 at genus level by the classifier (query type B, 163 ASV). The number of relevant sequences in the training set corresponding to each query might be expected to influence classifier confidence, i.e. having few representatives of a taxon may lead to low confidence and thus queries belonging to a poorly represented taxon are more likely to receive low confidence scores. Conversely, having many closely related sequences representing a taxon might also result in low confidence values because the classifier cannot distinguish between several likely matches. This latter scenario likely explains why some queries were not classified with confidence as themselves in our initial testing. To investigate both possible explanations for the different qualities of classification results for ASV that were ≥90% identical to something in the database by wateredBLAST but not classified at the species rank, we determined the number of records in cpnDB corresponding to the putative identities of the Type A and B queries (Table 1). Interestingly there was no significant difference between the average number of records per genus for Type A or B queries (Mann–Whitney, *P* = 0.0668), suggesting that the confidence of classification is not completely dependent on the number of representatives in the database and is likely affected by a combination of other factors including the characteristics of the individual sequences.

**Table 1:** Database coverage for saliva ASV queries that were ≥90% identical their nearest database neighbor by wateredBLAST, but classified at species level with confidence <0.8.

			cpnDB records per genus			
Query type	No. of ASV	No. of genera	Min	Max	Median	Average
Type A	887	26	1	213	18.5	46.2
Type B	163	23	1	211	9	26.7

Type A queries were classified with confidence >0.8 at the genus rank, whereas Type B queries were classified with confidence <0.8 at genus rank.

### Determining a classifier threshold to filter out non-cpn60 sequences

All “universal” primer PCR amplifications of marker genes will result in some level of non-target amplification, and the amount of those contaminating sequences generated will be influenced by factors including overall target abundance and the presence of host DNA in the case of host-associated microbiome samples [40]. How these off-target sequences are handled in the bioinformatic pipeline following microbiome amplicon library sequencing is variable, and many studies do not include a distinction between true target sequences that are “unidentified” and non-target sequences. Extensive analysis of cpn60 ASV sequences from previous microbiome studies and observations of cpn60 sequences from prokaryotes and eukaryotes suggest that the minimum similarity between any two known cpn60 sequences is ~55% [12, 40].

All the ASV with ≥55% identity to a sequence in cpnDB also had a phylum rank classification with >0.8 confidence, however, not all ASV with a phylum rank confidence <0.8 were <55% identical to a cpnDB sequence. This result demonstrates that confidence <0.8 at the phylum rank is not an ideal classifier-based criterion for identification of non-cpn60 sequences since it would filter out true cpn60 sequences. Visual inspection of classifier results from the microbiome ASV data suggested that a phylum rank confidence value of 0.6 was a reasonable alternative. To further evaluate these potential thresholds for identification of true cpn60 sequences, we performed a sensitivity and specificity analysis using percent sequence identity by wateredBLAST as the gold standard test. Sensitivity and specificity were calculated for both proposed threshold values for each of the human microbiome query sets (Table 2). Specificity was higher for phylum confidence of ≥0.8 than ≥0.6 in all cases, but sensitivity was higher for ≥0.6 than ≥0.8, indicating that using a confidence of ≥0.8 at the phylum rank would be more likely to result in screening out true cpn60 sequences. In microbiome studies aimed at cataloging diversity, sensitivity should be prioritized over specificity since this reduces the chances of novel cpn60 sequences being screened out (false negatives), therefore 0.6 at phylum rank is recommended for filtering cpn60 ASV data.

**Table 2:** Sensitivity and specificity of two potential classifier cutoffs for identification of true cpn60 sequences (defined as ≥55% identical to nearest database neighbor by wateredBLAST).

Classifier criterion		Saliva	Stool	Vagina	Average
Phylum confidence ≥0.6	Sensitivity	0.853	0.974	0.913	0.913
	Specificity	0.977	0.990	0.998	0.988
Phylum confidence ≥0.8	Sensitivity	0.788	0.947	0.826	0.895
	Specificity	0.993	1.00	0.999	0.996

**Fig. 3:**
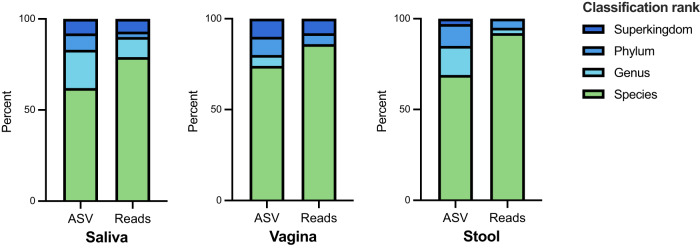
Percentage of cpn60 ASV sequences and proportion of the microbiome (percent of read counts across all samples) classified with a confidence level of >0.8 to the rank of species, genus, phylum and superkingdom. Results are shown for saliva (4960 ASV from 89 samples, 5.8 M reads), vagina (967 ASV from 283 samples, 1.5 M reads), and infant stool (2648 ASV from 584 samples, 2.4 M reads) microbiomes.

Once a cut-off was established, the results from the classifier for all three human microbiome data sets were re-examined with non-cpn60 sequences filtered out (Fig. 3). Filtration using the phylum confidence ≥0.6 criterion retained 4960/6737, 967/1950, 2648/4153 ASV sequences in the saliva, vagina and infant stool microbiome data sets, respectively. In all three environments, the majority of 150 bp ASV queries were classified confidently at species level: 62%, 74% and 69% of saliva, vagina and stool ASV, respectively (Fig. 3). When the relative abundance of the ASV in the microbiome data sets was considered, species level classification was achieved for 79%, 86% and 92% of the ASV observations (read counts) in the saliva, vagina and infant stool microbiomes, indicating that the more poorly classified sequences were also much less abundant in these microbiomes (Fig. 3). Results varied slightly among the three environments, with a larger proportion of the vagina and stool derived ASV sequences classified at the species rank compared to ASV sequences from the saliva microbiome, likely reflecting the representation of these environments in cpnDB and the extent to which organisms from these environments have been characterized and named. In fact, several of the most abundant but poorly classified ASV from the saliva microbiome were identical to unidentified cpn60 sequences from metagenomic studies of the oral microbiome.

### Effect of training sequence length on classifier results

The developers of the RDP classifier recommend using training sequences corresponding in length to the queries [45]. For example, if the data to be classified is V3-V4 of the 16 S rRNA gene, better accuracy will be obtained if the database of full-length 16 S rRNA gene sequences is trimmed to include only the V3-V4 region prior to training. We tested whether keeping the training set the same length as the queries would affect the results of the cpn60 classifier. We classified 150 bp stool microbiome ASV using the classifier trained on either 150 bp or full-length training sequences. Species rank confidence values were compared for each ASV and the results were highly correlated (Spearman’s correlation test, r = 0.9213, *p* < 0.0001) suggesting no significant difference in performance when the training sequences were trimmed to the length of the queries. Typical 16 S rRNA amplicon sequencing targets short variable regions that may account for a small proportion of the length of the 16 S rRNA gene. For example, the V3 region is ~175 bp (~12%) of the ~1.5 kb 16 S rRNA gene [46], compared to the 150 bp cpn60 sequence that accounts for ~27% of the ~555 bp “universal target” region of cpn60. In addition, the presence of multiple conserved and variable regions within the 16 S rRNA gene sequence (compared to the more uniform diversity across the cpn60 target [1]) may present a further obstacle for the classifier in cases where multiple variable regions and the intervening conserved regions are included in the amplicon.

### Classification of vaginal microbiome ASV in QIIME2

QIIME 2 [42] is one of the most widely used analysis platforms for microbiome amplicon sequence data. To demonstrate that classification of cpn60 sequences can be integrated into a QIIME2 workflow, we classified the vaginal microbiome ASV data using the q2-feature-classifier plugin. While the RDP classifier returns confidence values at every rank, the naïve Bayes classifier option in the q2-feature-classifier returns confidence values only for the rank reported. Of the vaginal microbiome ASV (*n* = 1950) classified, 821 were assigned to species, 24 were assigned to genus, 901 were assigned at higher ranks, and 204 were unassigned. As expected, the species assignments made by the q2-feature-classifier agreed with those from the RDP classifier (Supplementary File 1).

In conclusion, the RDP Classifier accurately classified reference cpn60 data, and provided rapid species rank classification for 150 bp ASV accounting for 79%, 86% and 92% of three different human microbiome data sets. We also demonstrated the cpn60 sequence classification can be easily integrated into a QIIME2 workflow, improving the accessibility of cpn60 amplicon sequencing methods to diverse users. The proportion of ASV classified to species rank will no doubt be improved with continuing expansion of cpnDB, by strategic enrichment of reference data for microbiomes of interest, and continuing efforts to characterize and name novel bacteria from diverse environments. Future work should include evaluation of additional classifiers for cpn60 sequence data.

## Supplementary information

Supplemental File 1

## Data Availability

The cpn60 sequences and taxonomy information (training data) and the trained classifier used in the performance evaluation described in this manuscript are available from Figshare DOI:10.6084/m9.figshare.21972278. Additional training data, scripts, and input files for training the q2-feature-classifier plugin for use in QIIME2 are available for download at https://github.com/HillLabSask/cpn60-Classifier.
